# Identification of genes and pathways associated with aluminum stress and tolerance using transcriptome profiling of wheat near-isogenic lines

**DOI:** 10.1186/1471-2164-9-400

**Published:** 2008-08-27

**Authors:** Mario Houde, Amadou Oury Diallo

**Affiliations:** 1 Centre TOXEN, Département des Sciences biologiques, Université du Québec à Montréal, C.P. 8888, Succ. Centre-ville, Montréal QC, H3C 3P8, Canada

## Abstract

**Background:**

Aluminum is considered the most limiting factor for plant productivity in acidic soils, which cover large areas of the world's potential arable lands. The inhibition of root growth is recognized as the primary effect of Al toxicity. To identify genes associated with Al stress and tolerance, transcriptome analyses of four different wheat lines (2 Al-tolerant and 2 Al sensitive) that differ in their response to Al were performed.

**Results:**

Microarray expression profiling revealed that 83 candidate genes are associated with Al stress and 25 are associated with tolerance. The stress-associated genes include important enzymes such as pyruvate dehydrogenase, alternative oxidase, and galactonolactone oxidase, ABC transporter and ascorbate oxido-reducatase. The Al tolerance-associated genes include the ALMT-1 malate transporter, glutathione S-transferase, germin/oxalate oxidase, fructose 1,6-bisphosphatase, cysteine-rich proteins, cytochrome P450 monooxygenase, cellulose synthase, zinc finger transcription factor, disease resistance response protein and F-box containing domain protein.

**Conclusion:**

In this survey, we identified stress- and tolerance-associated genes that may be involved in the detoxification of Al and reactive oxygen species. Alternative pathways could help maintain the supply of important metabolites (H_2_O_2_, ascorbate, NADH, and phosphate) needed for Al tolerance and root growth. The Al tolerance-associated genes may be key factors that regulate these pathways.

## Background

Aluminum is considered as the most limiting factor for plant productivity in acidic soils. It is estimated that over 50% of the world's potential arable land surface is composed of acid soils mostly distributed in developing countries [1,2]. Al tolerance is second to drought tolerance for its importance as agronomic trait for worldwide crop production. The root apex is considered the first target of Al toxicity and the reduction in root biomass leads to poor uptake of water and nutrients [3]. At the cellular level, Al toxicity results from a broad spectrum of deleterious effects caused by the Al^3+ ^ion, which is considered the most toxic species of Al for both plant and animal cells under low pH conditions [4]. It was proposed that the toxic effect of Al can be reduced by chelating Al in the rhizosphere with organic anions. Exudation of a variety of organic anions such as malate, citrate, or oxalate upon exposure to Al have been reported [2,5,6] and Al tolerance of several species can be enhanced by increasing organic acid biosynthesis [7-10]. Overexpression of the Al-inducible malate transporter improves Al tolerance in barley [11] while overexpression of the *Sb*MATE protein, a putative citrate transporter, improves Al tolerance in *Arabidopsis *and wheat [12]. The release of phosphate was also associated with Al tolerance, indicating that multiple mechanisms are involved in wheat [13]. In *Arabidopsis*, the major Al tolerance quantitative trait loci (QTL) was proposed to be proximal to the *AtALMT1 *malate transporter and to involve additional genes [14]. Fine mapping indicated that Al tolerance is more closely associated with a chromosome location that is distal to *AtALMT *by 1400 to 2100 kbp indicating that other gene products may regulate or complement its activity [14]. This regulation may be controlled at the transcriptional, translational, post-translational and enzymatic levels. The association of organic acid release and Al tolerance is not universal as several Al-sensitive plants were found to secrete large amounts of organic acids [15,16]. These results suggest that organic acid excretion, per se, is not a tolerance (or resistance) mechanism, but a consequence of biochemical reactions required for Al tolerance. In *Arabidopsis*, there was no induction of *AtALMT1 *by Al^3+ ^in the *stop1 Arabidopsis *mutant suggesting that the STOP1 zinc finger protein is required to activate *ALMT1 *transcription [17]. The *ALS3 *gene encodes an ABC transporter-like protein that is required for Al tolerance/resistance and may function to redistribute accumulated Al away from sensitive tissues to protect growing roots from Al toxicity [4]. Several genes up-regulated during Al exposure have been identified using various molecular approaches [18-22]. Overexpression of some of these genes in transgenic plants has resulted in modest improvement of Al tolerance, suggesting that they alleviate part of the toxicity caused by Al [23-27]. In rice, Al tolerance appears to be a complex multigenic trait that involves all twelve chromosomes. However, fewer loci were reported to be involved in other grasses [2,28,29]. Two major loci on chromosome 4DL and 6A, and additional loci with additive effects are involved in Al tolerance of the wheat cultivar Atlas66 [30,31]. The availability of near-isogenic lines derived from the cultivar Atlas66 having similar QTLs for Al tolerance provides a useful tool to identify genes associated with Al stress and tolerance [30,31].

In this study, several genes associated with Al stress and tolerance were identified using transcriptome analyses. The putative functions of identified genes in several biochemical pathways are discussed in relation to stress responses and the maintenance of root growth.

## Results

To identify genes associated with Al stress and tolerance in wheat, a large scale expression profiling study was initiated using the Affymetrix GeneChip^® ^Wheat Genome Array which allows the screening of 55052 transcripts. To better discriminate between genes that are associated with Al stress and tolerance, four different wheat lines (two Al-tolerant and two Al-sensitive) were analyzed. In addition to the well studied tolerant wheat Atlas66 and the sensitive wheat Bounty used in our previous study [19], we used two near isogenic lines (NILs) derived from a cross between Atlas66 and the sensitive cultivar Century [32]. The sensitive NIL OK91G108 (named Century-S thereafter) has a high degree of genetic similarity (96.9%) with the tolerant NIL OK91G106 (named Century-T thereafter). The major difference between tolerant and sensitive plants is the ability to maintain growth under high Al concentrations. The tolerant cultivars used in this study are able to grow for several days (50% rate of control plants or a root growth inhibition (RGI) = 50%) in the presence of 50 μM Al (result not shown). The sensitive cultivars are unable to grow at this high Al concentration and an RGI of 50% is obtained in the presence of 5 μM Al. We have previously shown that the stress-associated genes are expressed at comparable levels in the sensitive and tolerant cultivars after 24 hours when they are exposed to Al concentrations resulting in a similar RGI [19]. The microarray experiments were thus designed to identify genes that are differentially expressed after 24 hours of Al exposure at concentrations resulting in 50% RGI for all lines used.

### Microarray analyses

The number of Al-regulated genes (differential expression between Al-treated and untreated plants) is over 1000 when cultivars are analyzed separately. However, only 263 genes are differentially expressed in all four cultivars. ANOVA analyses with stringent p values retained 83 of these genes as highly significantly regulated (Table 1; additional file 1). Among these genes, 20 were previously identified as up-regulated by Al (references in Table 1). We randomly selected 4 of the stress candidate genes for qRT-PCR validation (see bold probeset IDs in Table 1). Two of them (genes # 2 and 3) are strongly over-expressed in response to Al, one (gene # 5, oxalate oxidase) was previously shown to be up-regulated by Al and the fourth one (gene # 49) was represented by 5 different genes (genes # 49, 61, 73, 74, and 76).

**Table 1:** Aluminum stress-regulated genes from wheat identified by microarray profiling

Gene number/ Response type^a^	ProbesetIDs^b^	Tentative annotation^c^	Representative GenBank/ Closest TIGR TC ID	Reference/ fold change^d^
1- Stress	Ta.25382.1.S1_at	Putative glutathione S-transferase	CD452690/TC271794	[20]/11.4
2- Pathogen	**Ta.192.1.S1_at**	WCI-5	U32431.1/TC236905	8.4
3- Unknown	**Ta.12921.1.S1_x_at**	Similar to XP_467711.1	CA601406/TC239727	7.9
4- Stress	Ta.5024.1.S1_x_at	Wali6	L28009.1/TC252513	[64]/7.3
5- Stress	**Ta.5557.1.S1_x_at**	Putative germin/oxalate oxidase	CD869243/TC250530	[19,65]/7.0
6- Unknown	Ta.12671.1.S1_a_at	Moderately similar to NP_909983.1	CK194385/TC265258	6.3
7- Unknown	Ta.8907.1.S1_at	Weakly similar to XP_481678.1	CD871652/TC257163	6.1
8- Pathogen	Ta.14071.2.S1_a_at	Putative nodulin MtN21	CA642218/TC236471	6.0
9- Unknown	Ta.13875.1.S1_at	Putative cyclin-dependent kinase 5 activator 2 precursor	AL830660/TC254582	5.9
10- Pathogen	Ta.231.1.S1_x_at	Secretory protein (WAS-2)	AF079526.1/TC234692	5.8
11- Pathogen	Ta.2784.1.A1_at	Chi 1 mRNA for chitinase 1	AB029934.1/TC251831	5.7
12- Unknown	Ta.25140.1.S1_at	Moderately similar to XP_477866.1 integral membrane protein-like	CD866293/TC268024	5.6
13- Stress	Ta.29814.1.S1_at	Class III peroxidase 15 precursor	CK213308/TC248113	[19]/5.5
14- Signalling	Ta.11671.1.S1_at	Putative heat shock transcription factor	BQ170572/TC268778	5.4
15- Stress	Ta.21267.1.S1_s_at	Wali3	CA694095/TC250582	[18]/5.4
16- Unknown	TaAffx.7032.1.S1_at	Unknown	CA670339	5.4
17- Unknown	Ta.14129.1.S1_at	Weakly similar to NP_181673.1 proline-rich family protein	BQ789170/TC253690	5.3
18- Stress	TaAffx.27822.1.S1_at	Wali5	CA669899/TC234419	[18]/5.3
19- Stress	Ta.22673.1.S1_s_at	Germin GF-2.8 precursor; oxalate oxidase	M21962.1/TC255645	[19]/5.3
20- Metabolism	TaAffx.118543.1.A1_at	Putative phragmoplastin (Oryza sativa)	BJ320269/TC288239 (rice)	5.2
21- Stress	Ta.962.1.A1_at	Class III peroxidase 15 precursor	BQ160711/TC247326	[19]/5.2
22- Stress	Ta.2793.1.S1_at	Tamdr1 mRNA; multidrug resistance protein 1 (ABC transporter)	AB055077.1/TC275845	[21]/5.1
23- Pathogen	Ta.231.1.S1_at	Secretory protein (WAS-2)	AF079526.1/TC234692	5.1
24- Stress	Ta.28233.1.S1_at	Putative iron/ascorbate-dependent oxidoreductase	CA599187/TC246980	5.1
25- Stress	Ta.18203.1.S1_at	Blue copper-binding protein	AF031195.1/TC265708	[20]/5.0
26- Unknown	Ta.975.2.S1_at	Moderately similar to XP_475934.1	CK217120/TC246830	5.0
27- Unknown	Ta.30765.1.S1_at	Weakly similar to XP_479604.1	CN011347/TC257484	5.0
28- Pathogen	Ta.8574.2.A1_at	Putative xylanase inhibitor TAXI-III	BQ162077/TC249173	4.8
29- Stress	Ta.24553.1.A1_at	Oxalate oxidase precursor (OXO1 gene)	CA662341/TC264833	[19]/4.7
30- Stress	Ta.11025.1.A1_at	FAD-binding domain-containing protein; putative arabinono-lactone oxidase (D-erythroascorbate)	BQ168402/TC235458	4.7
31- Unknown	Ta.5824.1.S1_s_at	Moderately similar to XP_467711.1	CA693256/TC263060	4.7
32- Metabolism	Ta.653.1.S1_at	Putative xyloglucan endo-1,4-beta-D-glucanase	CK196945/TC253973	[66]/4.6
33- Unknown	TaAffx.42638.1.S1_at	Unknown	BQ802968/TC253690	4.5
34- Pathogen/signalling	Ta.4479.1.S1_at	Putative disease resistance protein; leucine-rich repeat family protein	CN011595/TC262862	4.4
35- Stress	Ta.21350.1.S1_x_at	Wali5	L11882.1/TC234419	[18]/4.4
36- Stress	Ta.27945.1.S1_x_at	ABA responsive protein; putative glucosyltransferases (pfam domain) (cell wall metabolism)	CK216098/TC248224	4.3
37- Pathogen/signalling	Ta.4479.2.S1_a_at	Putative disease resistance protein; leucine-rich repeat family protein/putative protein kinase	CA735391/TC262862	4.3
38- Unknown	Ta.15199.1.S1_at	Weakly similar to NP_911238.1	CA653051/TC260490	4.3
39- Stress	Ta.30711.1.S1_x_at	Wali5; putative proteinase inhibitor (wrsi5-1)	AY549888.1/TC234467	[18]/4.3
40- Signalling	Ta.30908.1.S1_at	Putative EF-hand Ca2+-binding protein CCD1	CN013064/TC254944	3.9
41- Signalling	Ta.19062.1.S1_at	Putative EF-hand Ca2+-binding protein CCD1	CA646724/TC254944	3.8
42- Pathogen	TaAffx.15836.1.S1_at	Harpin induced protein pfam domain, hypersensitive response	BQ803322/TC300996 (rice)	3.7
43- Signalling	TaAffx.110751.1.S1_s_at	Putative EF-hand Ca2+-binding protein CCD1	CA685090/TC254944	3.7
44- Stress	Ta.6797.1.A1_at	Putative S-adenosylmethionine decarboxylase 2	BJ311153/TC261365	3.7
45- Stress	Ta.105.1.S1_at	Wali3	L11881.1/TC268347	[18]/3.6
46- Metabolism	Ta.4199.1.S1_a_at	Putative arabinoxylan arabinofuranohydrolase	CK199568/TC234741	3.5
47- Unknown	Ta.4343.1.S1_x_at	Weakly similar to NP_196028.2	CK212675/TC251862	3.5
48- Unknown	Ta.30942.1.S1_a_at	Unknown	BE515762/TC255181	3.4
49- Pathogen	**Ta.392.2.S1_at**	B2 protein; DCD (development and cell death) Interpro domain IPR013989	CA659319/TC234641	3.4
50- Signalling	TaAffx.128621.1.S1_at	Putative EF-hand Ca2+-binding protein; calmodulin-like	CK200510/TC269114	3.4
51- Metabolism	Ta.6099.1.S1_at	Nitropropane dioxygenase-like (pfam domain)	CA667895/TC235667	3.4
52- Pathogen	Ta.13701.1.S1_at	Benzoyl coenzyme A: benzyl alcohol benzoyl transferase; Putative hypersensitivity-related (hsr)protein	BQ280458/TC270357	3.4
53- Unknown	TaAffx.10772.1.A1_at	Unknown	CK213044/TC241105	3.4
54- Signalling	Ta.15067.1.S1_at	Putative EF-hand Ca2+-binding protein CCD1	CD876309/TC260557	3.4
55- Signalling	Ta.15067.1.S1_x_at	Putative EF-hand Ca2+-binding protein CCD1	CD876309/TC260557	3.4
56- Unknown	Ta.24654.1.S1_at	Unknown	CA605778/TC252436	3.4
57- Unknown	Ta.14221.1.S1_s_at	Weakly similar to XP_470656.1	BQ838103/TC258780	3.3
58- Unknown	Ta.21307.1.S1_x_at	Putative peroxidase	CK199589/TC249039	[19]/3.3
59- Unknown	TaAffx.22704.1.S1_at	Putative GABA-specific permease	CA746306/TC314548 (rice)	3.2
60- Signalling	Ta.8914.1.S1_at	Putative serine/threonine protein kinase	BQ162624/TC248336	3.2
61- Pathogen	Ta.392.2.S1_x_at	B2 protein; DCD (development and cell death) Interpro domain IPR013989	CA659319/TC234641	3.1
62- Metabolism	Ta.21166.1.S1_at	Putative shikimate kinase chloroplast precursor	CD492089/TC236265	3.1
63- Signalling	TaAffx.10874.1.S1_at	Putative Receptor protein kinase-like protein	CA609878/TC238909	3.1
64- Unknown	TaAffx.28156.1.S1_at	Unknown	CA664383/TC263848	3.1
65- Metabolism	Ta.29951.1.S1_at	Putative pyruvate dehydrogenase E1 alpha subunit, mitochondrial	CD903633/TC249785	3.0
66- Signalling	TaAffx.110751.1.S1_x_at	Putative EF-hand Ca2+-binding protein CCD1	CA685090/TC254944	3.0
67- Unknown	TaAffx.10772.1.A1_s_at	Unknown	CK213044/TC241105	2.9
68- Unknown	Ta.9255.1.S1_at	Weakly similar to NP_523812.1; Leucine-rich protein	CK208205/TC265940	2.9
69- Unknown	Ta.12565.3.S1_a_at	Putative ubiquitin-associated (UBA) protein	CA705730/TC247679	2.8
70- Stress	Ta.24150.1.S1_at	Glutathione-S-transferase 19E50	AY064481.1/TC266491	[20]/2.8
71- Pathogen	Ta.13785.1.S1_at	Xylanase Inhibitor Protein (Xip-I)	CK198324/TC264928	2.7
72- Metabolism	Ta.10549.1.A1_x_at	Alternative oxidase 3	CA609877/TC267601	[67]/2.7
73- Pathogen	Ta.392.1.S1_at	B2 protein; DCD (development and cell death) Interpro domain IPR013989	CK200130/TC234641	2.7
74- Pathogen	Ta.392.3.A1_s_at	B2 protein; DCD (development and cell death) Interpro domain IPR013989	CK164089/TC234641	2.7
75- Unknown	Ta.4674.1.S1_s_at	Unknown	BJ269896/TC243760	2.6
76- Pathogen	Ta.392.1.S1_x_at	B2 protein; DCD (development and cell death) Interpro domain IPR013989	CK200130/TC234641	2.6
77- Metabolism	Ta.4199.2.S1_at	Putative arabinoxylan arabinofuranohydrolase	CA601651/TC234741	2.6
78- Unknown	Ta.28879.1.S1_at	Protein phosphatase type 2C	BJ306387/TC263663	2.5
79- Unknown	Ta.28879.2.S1_x_at	Protein phosphatase type 2C	CA744010/TC263663	2.4
80- Unknown	Ta.25514.1.S1_s_at	Polygalacturonase activity (GO:0004650)	CA663409/TC269433	2.3
81- Unknown	Ta.12879.1.S1_at	Moderately similar to XP_463452.1	CD490901/TC241688	0.3
82- Pathogen	Ta.7963.2.S1_x_at	Putative disease resistance response protein	CK215257/TC251022	0.2
83- Stress	Ta.22968.1.S1_at	Putative lipid transfer protein	CA614519/TC247557	[67]/0.2

a: The response type is based on previous publications.

b: According to Affymetrix Gene Chip^® ^wheat genome array description. The probesetIDs are presented in decreasing order of differential expression (most over-expressed to most down-regulated). The last 3 entries are down-regulated genes. For more details on gene expression level, see Additional file 1. Genes subsequently tested by qRT PCR are in bold.

c: Annotations were made based on Affymetrix gene annotation complemented with BLAST results using the public representative ID provided with the Affymetrix Gene Chip^® ^wheat genome array.

d: The fold change represents the mean ratio of gene expression in all cultivars exposed to Al (50% RGI)/the controls not treated with Al.

As found for stress-associated genes, microarray analyses allowed the identification of more than 1000 genes potentially associated with Al tolerance when selection was performed using only one pair of wheat lines (differential expression between Atlas66/Bounty or Century-T/Century-S exposed to Al) but this number was reduced to 69 common genes when both pairs were considered. This result suggests that a large number of genes are associated with cultivar specific responses and only a limited number of them are associated with Al tolerance. This list was reduced to 25 genes using ANOVA analyses with stringent p values (Table 2; additional file 2). These genes were subdivided into constitutively or differentially expressed genes based on the difference in expression between Al-treated and non-treated control plants. Table 2A lists the 6 constitutively expressed genes classified in decreasing order of expression (average fold expression of the two tolerant compared to the two sensitive cultivars). The most strongly expressed gene is the previously identified Al malate transporter ALMT-1 [33]. We selected two of these genes for qRT-PCR validation (gene # 84 encodes ALMT-1 and is more expressed in tolerant cultivars while gene # 89 encodes a protein containing an F-box domain and is more expressed in sensitive cultivars; Table 2A). Table 2B lists the 19 genes that are regulated by Al and differentially expressed between the two tolerant and the two sensitive cultivars. Four of these genes were selected for validation by qRT-PCR (see bold probeset IDs in Table 2B).

**Table 2:** Genes associated with Al tolerance in wheat, as identified by microarray profiling

**A: Constitutively expressed genes associated with Al tolerance.**				
Gene number response type^a^	Probeset IDs^b^	Annotation^c^	Representative GenBank/ Closest TIGR TC ID	Reference/fold change^d^

84- Stress^a^	**Ta.30659.1.S1_at**	Almt1-1 mRNA for aluminum-activated malate transporter	AB081803.1/TC275842	[33]/5.4
85- Unknown	TaAffx.11437.1.S1_at	Unknown	CD916477/TC237550	3.1
86- Unknown	Ta.6595.1.S1_at	Weak similarity to pinin	CA680476/TC239245	0.4
87- Metabolism	Ta.9190.2.S1_at	Putative Anaphase promoting complex subunit 10	BJ321280/TC237861	0.3
88- Unknown	Ta.7249.1.S1_at	Unknown	CA713200/TC238862	0.2
89- Signalling	**TaAffx.16664.1.A1_at**	F-box containing domain (IPR001810) and F-Box associated domain (IPR006527); Ubiquitination	CK205523/TC255586	0.2

**B: Up-regulated genes associated with Al tolerance.**				

Response	Probeset IDs^a^	Annotation^a^	Representative GenBank/ Closest TIGR TC ID	Reference/fold change^d^

90- Unknown	**Ta.23271.1.S1_s_at**	unknown	CA680274/TC249675	7.6
91- Stress	**Ta.8545.1.S1_at**	Glutathione S-transferase (GST)	BQ162041/TC259746	5.7
92- Pathogen	**Ta.21314.1.S1_x_at**	Similar to disease resistance response protein	CA669694/TC266782	5.2
93- Unknown	TaAffx.26343.1.S1_at	Unknown	CA689752/TC257163	4.9
94- Metabolism	Ta.8447.1.S1_a_at	Putative cytochrome P450 monooxygenase	CA669038/TC236876	[67]/4
95- Pathogen	**Ta.24632.1.S1_at**	Pathogen response serine-type endopeptidase inhibitor activity; putative protease inhibitor	BE405372/TC248320	4
96- Stress	Ta.3118.1.S1_at	Glutathione S-transferase	BE515437/TC238392	3.2
97- Unknown	TaAffx.86317.1.S1_at	Yippee-like protein IPR004910; role in cell division	CA611222/TC268232	3.2
98- Pathogen	Ta.24632.1.S1_x_at	Pathogen response serine-type endopeptidase inhibitor activity; putative protease inhibitor	BE405372/TC248320	3.1
99- Unknown	Ta.10326.1.S1_at	Unknown	BJ244180/TC238059	3.1
100- Unknown	Ta.14224.1.S1_at	Weak similarity to Protamine 1B or Zinc Knuckle domain	CK214385/TC252792	3.1
101- Metabolism	Ta.28890.1.A1_s_at	Fructose-1,6-bisphosphatase isozyme 2 (F1,6-BP)	CA686703/TC235271	2.9
102- Pathogen	Ta.24632.1.S1_a_at	Pathogen response serine-type endopeptidase inhibitor activity; putative protease inhibitor	BE405372/TC248320	2.9
103- Unknown	Ta.13302.1.S1_at	Unknown	BQ801428/TC258348	2.5
104- Unknown	Ta.23097.1.S1_x_at	Weak similarity to Adhesive/ proline-rich-like protein	CA699090/TC241038	2.4
105- Unknown	Ta.29761.1.A1_at	Similar to At2g31940	AJ609795/TC243334	2.4
106- Metabolism	Ta.4084.1.S1_at	Putative cellulose synthase-like protein OsCslE1 (cell wall metabolism)	BJ264002/TC253821	2.4
107- Signalling	TaAffx.12876.1.S1_at	Putative C2H2 type zinc finger transcription factor	BJ220837/TC275754	2.1
108- Pathogen	Ta.7883.1.S1_x_at	Putative disease resistance response protein	CK212322/TC267223	0.2

a: The gene are numbered consecutively to Table 1 to simplify presentation in the text and Figure 3. The response type is based on previous publications.

b: According to Affymetrix Gene Chip^® ^wheat genome array description. The probesetIDs are presented in decreasing order of differential expression (most over-expressed to most down-regulated). The last 4 entries in Table 2A are more expressed in sensitive cultivars. The last entry in Table 2B is a down-regulated gene in the tolerant cultivars. For more details on gene expression level, see Additional file 2. Genes subsequently tested by qRT PCR are in bold.

c: Annotations were made based on Affymetrix gene annotation complemented with BLAST results using the public representative ID provided with the Affymetrix Gene Chip^® ^wheat genome array.

d: The fold change represents the mean ratio of gene expression in the two tolerant cultivar/the two sensitive cultivar (50% RGI).

### Quantitative RT-PCR validation of microarray data

**Figure 1:**
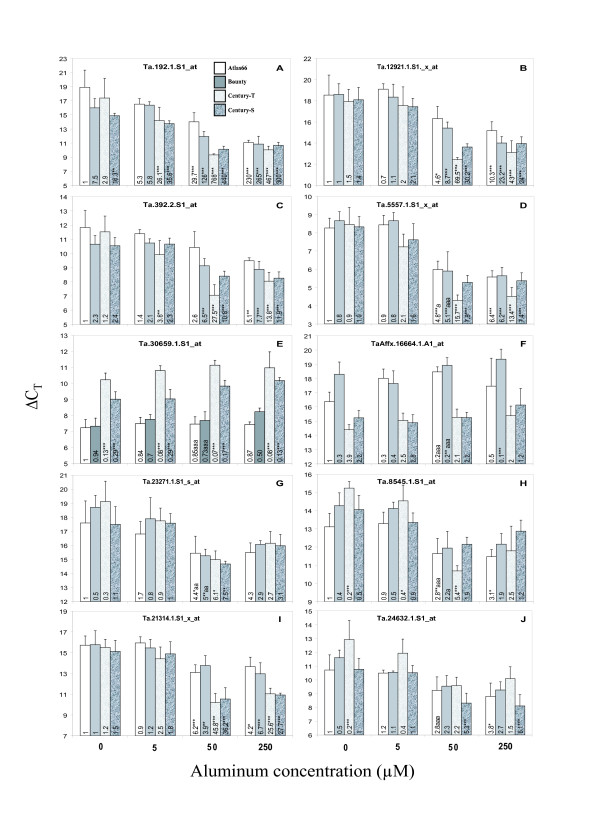
**Quantitative Real-Time PCR analysis of candidate genes**. Control non-treated plants were exposed to a solution of 1 mM CaCl_2_, pH 4.15 for 24 hours while Al treatment was performed in the same solution containing 5, 50 or 250 μM Al. qRT-PCR was performed on four biological replicates. RNA was extracted, reverse-transcribed and the expression of genes identified in Tables 1 and 2 was measured using qRT-PCR. The C_T _values were normalized using the 18S RNA (note that a lower C_T _means increased expression and a C_T _difference of 1 represents a two-fold difference in expression). A statistical difference between each sample and the expression observed in Atlas66 not exposed to Al is indicated by an asterisk in the histogram columns (*: p < .05; **: p < .01; ***: p < .001). A statistical difference between tolerant cultivars exposed to 50 μM Al and their sensitive counterpart exposed to 5 μM Al is indicated by an "a" after the asterisks (a: p < .05; aa: p < .01; aaa: p < .001).

We selected ten genes for validation of the microarray data by qRT-PCR (4 among the stress-associated candidates and 6 among the tolerance-associated candidates, as indicated above). Four different Al concentrations (0, 5, 50 and 250 μM Al) were used to determine the effect on gene expression. Four new replicates of each Al exposure conditions using all wheat lines were prepared. The values indicated in each histogram column represent the expression levels relative to the first column (cultivar Atlas66 not exposed to Al) (Fig. 1A–J). The level of expression of the tested genes under control conditions is generally similar in the tolerant and sensitive cultivars.

Genes were classified as associated with Al tolerance when there was a significant difference in expression in both tolerant cultivars (Atlas66 and Century-T exposed to 50 μM Al) compared to their respective sensitive counterparts (Bounty and Century-S exposed to 5 μM Al). These conditions allow the detection of either 1) Al-regulated genes that may be expressed differentially in tolerant and sensitive lines, or 2) genes not regulated by Al (constitutively expressed) but showing constitutive difference in expression between tolerant and sensitive lines. Once classified as associated with stress or tolerance, gene regulation by Al was confirmed by comparing the Al-treated and non-treated controls. qRT-PCR, analyses confirmed that three (Ta.192.1.S1_at, Ta.12921.1.S1_x_at and Ta.392.2.S1_at, Fig. 1A–C) of the four stress candidate genes are stress-associated while the 4^th ^gene (Ta.5557.1.S1_x_at, Fig. 1D) is more closely associated with Al tolerance. Among the six selected tolerance candidates genes, four were confirmed as associated with Al tolerance (Fig. 1E–H: Ta.30659.1.S1_at, TaAffx.16664.1.A1_at, Ta23271.1.S1_s_at, Ta.8545.1.S1_x_at) while the last two genes (Ta.21314.1.S1_x_at and Ta.24632.1.S1_at, Fig. 1I–J), were not clearly linked to either stress or tolerance.

**Figure 2:**
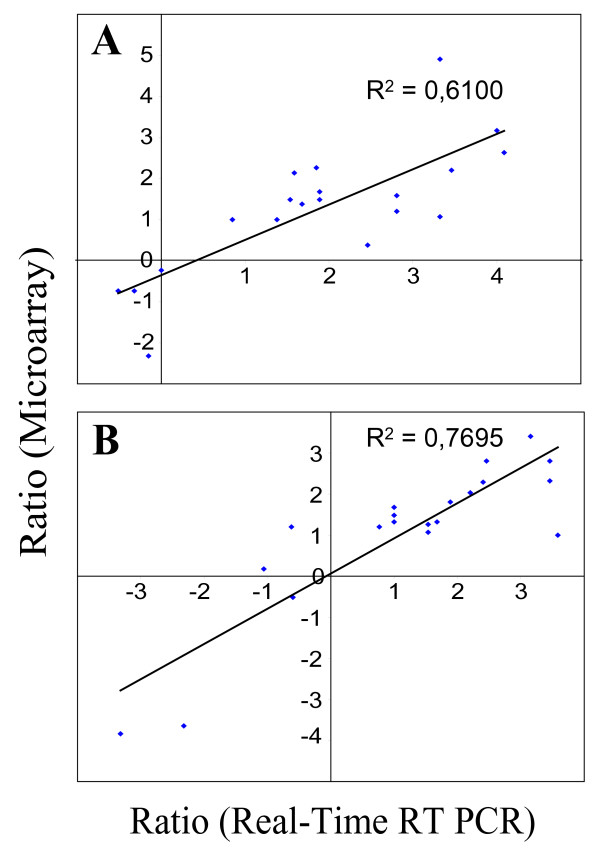
**Correlation of qRT-PCR and microarrray data**. A – The ratio of gene expression (log_2 _scale) in plants treated with Al and the control (not treated with Al) was calculated in the microarray experiments and plotted against the ratio calculated in the qRT-PCR analyses. B – The ratio of gene expression (log_2 _scale) in tolerant and sensitive cultivars exposed to Al giving the same RGI (Atlas66/Bounty or Century-T/Century-S) was calculated in the microarray experiments and plotted against the ratio calculated in the qRT-PCR analyses.

The correlation between gene expression data obtained by microarray and qRT-PCR is presented in Fig. 2. Al-regulated genes are shown in Fig. 2A for the cultivars Atlas66 and Bounty. The fold change in gene expression (Al-treated VS control) obtained using microarray experiments compared to the fold change obtained using qRT-PCR gives a correlation coefficient of 0.61. A similar analysis was performed to compare the change in gene expression between the tolerant and sensitive cultivars (Atlas66 50 μM Al/Bounty 5 μM Al and Century-T 50 μM Al/Century-S 5 μM Al) calculated from the microarray or qRT-PCR data (Fig. 2B). In this case, a correlation coefficient of 0.77 was obtained between the two experimental methods. These correlation coefficients are very good considering that microarray data are semi-quantitative and subject to error for multigene families where different transcripts could hybridize to similar probes on the array. qRT-PCR data are more specific since the amplification of single transcripts are confirmed by melting curves and gel analyses. The Ta.5557.1.S1_x_at was initially selected as a stress-associated gene by microarray profiling (gene no 5 in Table 1) while it was reclassified as associated with Al tolerance using qRT-PCR (Fig. 1D). This may be related to the presence of multiple oxalate oxidase genes in wheat. Based on these results, we conclude that most of the genes identified in Table 1 and Table 2 are associated with Al stress and tolerance. However, real-time PCR analyses will be required to validate the other genes that were identified using microarrays.

## Discussion

Several Al-associated QTLs with major and minor contributions were reported, indicating that Al tolerance is a multigenic trait. A large scale transcriptome analysis was initiated to identify some gene components of this multigenic trait. The genes that are differentially expressed between tolerant and sensitive cultivars are of particular interest since the tolerant cultivars are exposed to Al concentrations that are ten times higher, yet they are still growing at the same rate as the sensitive cultivars. This design allows the distinction of genes that are regulated in response to stress from those that are associated with Al tolerance. Transcriptome analyses allowed the identification of several genes with known function that could improve our understanding of the biochemical processes and pathways involved in Al stress and tolerance. As indicated in the result section, 20 of the 83 genes associated with the Al stress response were identified in previous studies indicating that the genes associated with Al stress are appropriately identified using this approach.

### Signal transduction

We have previously shown that Al inhibits a redox reaction associated with root growth and Al tolerance [34]. The perturbation of this redox reaction by Al could lead to the accumulation of different reactive oxygen species which can stimulate a redox signalling pathway and increase the expression of antioxidant enzymes [35]. The accumulation of reactive oxygen species during Al exposure was observed in maize, soybean and wheat [36-38]. Al was also shown to affect mitochondrial functions leading to ROS production [39]. Table 1 shows that the genes encoding WCI-5 (gene # 2), WAS-2 (genes # 10 and 23) and the EF-hand Ca^2+^-binding protein (genes # 40, 41, 43, 54 and 55) are up-regulated during Al exposure. In *Arabidopsis*, the *WCI-5 *and *WAS-2 *genes were shown to be induced via a MAP kinase pathway associated with the pathogen response [40]. In wheat seedlings, the *WCI-5 *gene was shown to be induced by pathogen infection [41] and mediated by the EF-hand Ca^2+^-binding protein CCD1 [42]. These results suggest that a MAP kinase pathway is stimulated by Al in wheat roots as observed in cell suspension cultures of *Coffea arabica *L. [43]. Three different protein kinase-like genes (genes # 37, 60, 63) are up-regulated during Al exposure suggesting that different signal transduction pathways may be activated. We identified two other potential signalling genes associated with Al tolerance. The first one encodes a putative C2H2 zinc finger transcription factor (gene # 107) that has a 33 aa region sharing 50% identity with the STOP1 C2H2 zinc finger protein required for Al^3+ ^induction of *ALMT1 *in *Arabidopsis *[17]. The other gene encodes an F-box containing protein (gene # 89), which is member of a family known for its involvement in the controlled degradation of target proteins. An *Arabidopsis *F-box protein homologous to a bacterial redox sensor was proposed to be involved in the cell wall receptor-like associated kinase (WAK1) signalling pathway [44]. Since WAK1 was shown to be transiently overexpressed during Al exposure and associated with Al tolerance [27], the reduced expression of an F-Box protein may stimulate this pathway by allowing a stronger expression of WAK1 in tolerant cultivars. The *TIR1 *gene is another F-Box protein in *Arabidopsis *that interacts with different proteins in the SCF^TIR1 ^complex to mediate responses to auxin [45,46]. These two genes (C2H2 zinc finger and F-Box) require further characterization and functional analysis to understand their roles in the response to Al.

### Management of Al-associated stresses and maintenance of energy supply

**Figure 3:**
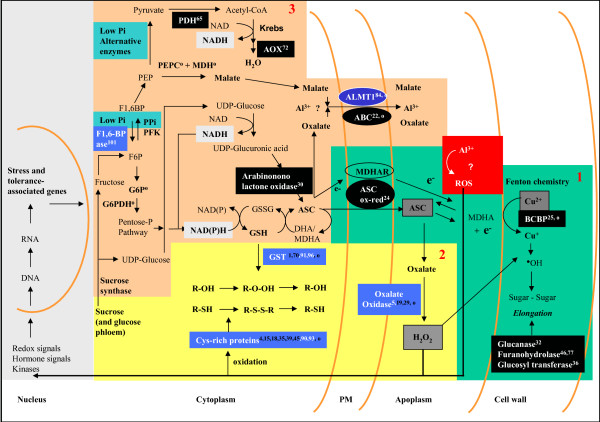
**Putative gene function in ascorbate homeostasis and Al tolerance**. Al tolerance-associated genes are represented by white numbers* in the dark blue boxes (numbers in black are other genes of the same type associated with Al stress). Genes associated with Al stress responses are in black boxes. **Box 1) **The normal process of cell wall loosening is promoted by the production of hydroxyl radicals (^•^OH) to break glycosidic linkages (sugar-sugar). The hydroxyl radicals are produced non-enzymatically by the Fenton chemistry in the presence of Cu^2+^, H_2_O_2 _and ascorbate which serves as electron donor (the three molecules are in grey boxes). In the apoplasm, ascorbate (ASC) is transformed to monodehydroascorbate (MDHA + e^-^) and a free electron is used to reduce the Cu^2+ ^to Cu^+ ^(needed for the Fenton reaction). An ascorbate oxido-reductase (ASC ox-red), or other plasma membrane transporters may be involved in the regeneration of apoplasmic ASC. Cytoplasmic ASC and NADH are needed to maintain the level of ASC in both compartments. Al (red box) was shown to block a redox reaction (electron transfer) [34] suggesting that it interferes either with the electron transfer from the cytoplasm to ASC (regeneration) or from ascorbate to Cu^2+ ^(utilization). The diverted electron may generate different ROS molecules that will cause oxidative stress and trigger a redox response and the induction of various genes. **Box 2) **A slower regeneration of ASC could lead to its degradation and to the accumulation of oxalate. The induction of oxalate oxidase can use this oxalate to maintain the production of H_2_O_2 _and support the Fenton reaction. If the apoplasmic ASC concentration decreases too much, H_2_O_2 _may not be used efficiently in the cell wall loosening process and will accumulate. This ROS can cause the oxidation of several targets including different cysteine-rich proteins associated with Al tolerance. GSTs associated with Al stress or tolerance can participate in the protection or repair of oxidized targets. **Box 3) **Mobilisation of phloem sugars and reduction in phosphate availability (Low Pi in green) caused by Al can also participate in gene regulation and stimulate alternative pathways to increase the availability of important metabolite precursors such as NADPH (in light grey) through the pentose-P pathway (G6P: Glucose-6 phosphate; G6PDH: glucose 6 phosphate dehydrogenase); and phosphate (F1,6BP, Fructose 1,6 bisphosphatase; PPi PFK: pyrophosphosphate dependent phosphofructokinase). Other alternative pathways are activated to maintain the Krebs cycle (PDH: pyruvate dehydrogenase, AOX: alternative oxidase) and to stimulate malate production (PEPC: phosphoenol pyruvate carboxylase; MDH: malate dehydrogenase). UDP-glucose can be used to generate ASC and NADH (arabinonolactone oxidase). Other genes such as ALMT1 or ABC transporters may be involved in Al chelation and transport to exclude Al. *: the numbers represent genes identified in this work; see Table 1 and Table 2. **o**: genes, enzyme activities or metabolite changes identified in other studies. For references, see text.

The identification of stress-regulated genes provide new tools to reduce Al stress, as shown by the ectopic overexpression of some Al stress-regulated genes [23-26]. Among the 83 candidate genes regulated by Al stress (Table 1), several could play a role in alleviating phosphate deficiency and provide energy to fight oxidative stress. Nutrient deficiency, and especially phosphate, occurs in the presence of Al due to the precipitation of Al-phosphate [47]. Under phosphate deficiency, several pathways are hindered due to the reduced availability of ATP and related nucleoside phosphates. These imbalances lead to the induction of alternative pathways of glycolysis to maintain energy and carbon skeletons for key metabolic processes [48]. The fructose-1,6-bisphosphatase (gene # 101) associated with Al tolerance (Table 2) is normally inhibited by phosphate but is stimulated under low phosphate availability (Fig. 3, Box 3). This activity produces fructose-6-P and liberates Pi. The glycolytic alternative enzyme pyrophosphate phosphofructokinase is stimulated by low phosphate and can resynthesise fructose-1,6-bisphosphate and liberate more Pi. This apparently futile cycle is useful to conserve ATP and recycle Pi that is readily available in the pyrophosphate molecule [48]. Furthermore, since sucrose is the most abundant photosynthate available in the phloem sap, fructose (from sucrose) is readily available and will enter the glycolytic pathway at the level of fructose-6-P thereby maintaining a stable supply of this metabolite. The high availability of fructose-6-P (and other hexose-P) can provide sufficient metabolites to support both the pentose phosphate pathway and organic acid synthesis (Fig. 3, Box 3). Al-tolerant rye was found to maintain a higher content of glucose-6-phosphate than Al-sensitive wheat in response to Al exposure [49]. This metabolite is an important precursor of the pentose phosphate pathway (with glucose-6-phosphate dehydrogenase (G6PDH) as the first enzyme) and is an important source of NADPH production. The importance of energy metabolism in modulating Al tolerance is exemplified in *Pseudomonas fluorescens *where G6PDH was shown to play a pivotal role in enhancing NADPH production and protecting against ROS toxicity [50]. The up-regulation of pyruvate dehydrogenase (gene # 65) and alternative oxidase (gene # 72) supports the involvement of an alternative pathway to maintain energy production (Fig. 3, Box 3) [48]. Members of the alternative oxidase gene family were found to be highly responsive to oxidative stress and to reduce mitochondrial ROS production while maintaining NADH supply [51,52]. In Al tolerant plants, a higher NADH supply (and NADPH through the pentose phosphate pathway) would provide reducing power to regenerate ascorbate and glutathione to fight oxidative stress (Fig. 3, Box 3).

Exudation of organic acids has received much attention as an Al tolerance (resistance) mechanism. The secretion of malate or other organic acids (citrate, oxalate) can chelate Al in the rhizosphere and has been associated with Al tolerance in several species [7-10,33]. Enzymes involved in malate synthesis (PEP carboxylase and malate dehydrogenase, Fig. 3 Box 3) were shown to be stimulated during Al stress [53,54]. These enzymes are also stimulated during Pi deficiency where malate is known to mobilize external Pi [48]. These two actions are complementary since the chelation of Al will also free Pi from precipitated Al-phosphate in the rhizosphere. Some of the genes identified in this study may complement the ALMT1 malate transporter to improve Al exclusion. An interesting possibility is that organic acids, such as malate or oxalate, could form intracellular Al-bound complexes that are transported outside the cell (Fig. 3, Box 3). It is possible that the ALMT1 malate transporter (gene # 84) associated with Al tolerance can transport free malate but that it would require a co-factor to transport the larger Al-bound malate complex. Oxalate is another organic acid known to be a strong Al chelator associated with Al tolerance in Taro [55]. This organic acid is another potential molecule that could form an Al complex transported out of the cell by an anion transporter such as ALMT1 or the ABC transporter (gene # 22) that is up-regulated during Al exposure.

Several proteins encoded by genes that are up-regulated during Al exposure have a high content of cysteine (Fig. 3, Box 2). The high amount of thiol groups in these proteins may help protect enzymes containing thiol sensitive sites by providing new targets of oxidation. The presence of 11 cysteine residues in the small protein (128 aa) encoded by gene # 90 may explain why this gene is associated with Al tolerance (Fig. 3, Box 2). The up-regulation of four different GSTs (genes # 1, 70, 91, 96), containing cysteine residues in their active sites, indicate that these enzyme may be inactivated by oxidative stress. The synthesis of new enzyme molecules during Al exposure may partially compensate for GST inactivation. On the other hand, two of the four GSTs are associated with Al tolerance (genes # 91, 96). These GSTs may help to reduce oxidative stress as suggested for *parB*, a tobacco GST isoform [25].

### Maintenance of root growth

The ability to maintain root elongation under Al stress may require complementary mechanisms such as Al sequestering (outside the cell, or Al exclusion) and increased expression of genes involved in detoxification or root growth. We identified several genes that could play a role in maintaining root growth. Different enzymes such as sugar hydrolases and transferases may participate in cell wall remodelling/loosening in the elongation zone (genes # 32, 36, 46, 77). During the cell wall loosening process, ascorbate provides an electron to reduce Cu^2+ ^to Cu^+ ^(Fig. 3, Box 1) [56,57], which participates in the Fenton reaction with H_2_O_2 _to ultimately produce •OH radicals. The non-enzymatic cleavage of cell wall polysaccharides thus requires the presence of three basic molecules: ascorbate, Cu^+2 ^and H_2_O_2 _(grey boxes in Fig. 3) [56,57]. The enzymatic reactions required to maintain/regenerate ascorbate and provide H_2_O_2 _are essential components to maintain root growth and are likely associated with Al tolerance. The redox activity previously shown to be associated with Al tolerance [34] may represent the transfer of electrons required to regenerate the apoplasmic ascorbate. The great avidity of Al^3+ ^for electrons (Fig. 3, Box 1) could prevent the regeneration of apoplasmic ascorbate and inhibit root growth. Oxalate oxidase may help maintain the production of H_2_O_2 _for the Fenton reaction (Fig. 3). However, H_2_O_2 _could become a substrate for cell wall peroxidases which are not inhibited when ascorbate levels are reduced [58]. The inhibition of peroxidase activity was associated with Al tolerance [59,60] and may help maintain H_2_O_2 _levels needed for the non-enzymatic wall loosening process. In our study, three peroxidases (genes # 13, 21, 58) are up-regulated and could be related to the reduced growth rate under our experimental conditions. Transcripts encoding blue copper binding proteins (BCBP) (gene # 25) is possibly up-regulated to stimulate the Cu^2+^-dependent Fenton reaction needed for cell wall loosening. When apoplasmic ascorbate loses an electron, it is transformed into monodehydroascorbate/dehydroascorbate (MDHA/DHA) and new electrons are needed to regenerate ascorbate and avoid degradation into oxalate (Fig. 3, Box 1). The up-regulation of an ascorbate dependent oxidoreductase (gene # 24) could be associated with the regeneration of apoplasmic ascorbate (Fig. 3, Box 3). Impaired ascorbate metabolism was proposed to be involved in the reduction of root growth in squash roots exposed to Al [38] while a higher level of ascorbate and gluthathione was shown to be associated with Al tolerance in tobacco [61]. Furthermore, feeding with D-glucose or L-galactono-γ-lactone to enhance ascorbate levels was able to improve Al tolerance in rice [62]. These results indicate that maintaining a high ascorbate level is an essential aspect of Al tolerance. Gene # 30 annotated as an arabinonolactone oxidase is homologous to galactonolactone oxidase involved in ascorbate synthesis (Fig. 3, Box 3). This last gene participates in the regeneration of cytoplasmic ascorbate while providing additional NADH (from glucose, Fig. 3, Box 3). The arabinonolactone oxidase is known to synthesize erythroascorbate in yeast. Interestingly, the overexpression of the yeast arabinonolactone oxidase enzyme in rice improves Al tolerance [62].

## Conclusion

Genome wide expression profiling and qRT-PCR using different wheat cultivars subjected to controlled stress treatments allowed the identification of several new genes associated with Al stress and tolerance. Several genes associated with Al tolerance could play an important role in maintaining the energy balance and Al exclusion. The maintenance of ascorbate homeostasis is proposed to be a key element to sustain elongation growth. The availability of genes associated with Al tolerance provides new tools for QTL analyses and for breeding programs aimed at improving Al tolerance of cultivated crops.

## Methods

### Plant material, growth and Al exposure conditions

Two wheat cultivars with a high tolerance to Al (*Triticum aestivum *L. cv. Atlas66 and the near isogenic line OK91G106, named Century-T in this work; [32]) and two wheat cultivars with low tolerance to Al (*T. aestivum *L. cv. Bounty and the near isogenic line OK91G108, named Century-S in this work; [32]) were grown as previously described, and treated under conditions where Al remains mostly in the Al^3+ ^form [19]. To reduce pH variations and ensure that Al speciation was stable throughout the experiment, at least 100 ml of solution was used for each plant. The root growth inhibition (RGI) is expressed as 100 × [1-(root growth of Al-treated seedling divided by the root growth of control seedlings)]. Replicate experiments were performed on different days with one series of Al concentration per day per cultivar in order to rapidly collect root tips.

### RNA isolation and microarray profiling

Root tips (5–10 mm) were isolated after 24 hours of exposure to Al and frozen on dry ice. Total RNA was isolated from the root tips (5 mm long) of 50 plants collected from the various genotypes using the RNeasy Plant Mini Kit (Qiagen). The RNA quality was assessed on agarose gels and with the Bioanalyzer 2100 (Agilent). Microarray profiling was performed according to Affymetrix protocols at the Functional Genomics Platform of McGill University and Génome Québec Innovation Centre using the Affymetrix GeneChip^® ^Wheat Genome Array. The microarray results were deposited in the database of ArrayExpress under the accession number E-TABM-454. Three biological replicates of the different lines were treated with Al concentrations resulting in 50% RGI: 50 μM for the tolerant cultivars Atlas66 and Century-T and 5 μM for the sensitive cultivars Bounty and Century-S. The wheat NILs Century-T and Century-S were used only to prepare the Al-treated plants due to the limited number of seeds available. In this case, the average value obtained for the untreated (not exposed to Al) Atlas66 and Bounty was used as the reference.

### Quantitative Real-Time PCR

The level of expression of selected genes was validated by Real-Time PCR. Probes were designed using the primer3 software and primer specificity was verified on agarose gels and by T_m _measurements at the end of the Real-Time PCR reactions. Total RNA was prepared (RNeasy Plant Mini Kit; Qiagen) from the root tips (5 mm long) of 50 plants collected from the various genotypes and reverse transcribed using SuperScript II reverse transcriptase and random hexamers (SuperSript™ First-Strand; Invitrogen). Quantitative Real-Time was performed on an ABI 7000 Real Time Cycler using the specific primers described in additional file 3. One-tenth dilutions of the cDNAs were used as template for the qRT-PCR in a total volume of 25 μL as follows: 12.5 μL SYBR Green (Platinum^® ^SYBR^® ^Green qPCR SuperMix-UDG, Invitrogen) 1.0 μL primer mix (50:50 mix of forward and reverse primers at 10 pmol/μL each) and 2 μL template. The reaction conditions were: 10 min at 95°C followed by 40 cycles of 1 min at 95°C and 30 s at 60°C.

### Data analyses

The microarray data were analyzed using the robust multi-array average (RMA) software (RMA version 0.2) of background adjusted, normalized, and log transformed perfect match values [63]. A two-fold cut-off value (log_2 _≥ 1) was arbitrarily set to indicate differential gene expression between two samples. Genes showing a differential expression greater than two-fold (RMA differential expression of log_2 _≥ 1) between the Atlas66 and Bounty controls were excluded from the reference set as these may represent inherited cultivar basal expression levels that could bias the analysis. Overall, this excluded 1.26% of the genes on the microarray (773 of 55,052) demonstrating that most genes are expressed at a similar level between the two cultivars. Genes that are differentially expressed to the same extent in all four cultivars exposed to Al (giving 50% RGI) compared to the non-treated cultivars were selected and classified as candidates for stress-associated genes. The differentially expressed genes between the two tolerant cultivars exposed to Al (Atlas66 and Century-T) and their respective sensitive counterparts (Bounty and Century-S) exposed to Al concentrations resulting in 50% RGI were classified as candidate genes associated with Al tolerance. These genes were subdivided in two groups (constitutively expressed or Al-regulated) based on the average level of regulation in the two tolerant cultivars. Genes were considered constitutively expressed when the differential signal between Al-treated and non-treated samples was less than two-fold (log_2 _< 1). An analysis of variance was performed using GraphPad InStat 3 to select genes that are differentially expressed under the conditions specified for each analysis.

For Quantitative Real-Time PCR analyses, the amplification efficiency (90% to 100%) for the different primer sets was determined by amplification of cDNA dilution series using 80, 20, 10, 5, 2.5, and 1.25 ng per reaction (data not shown). The variance (standard error) was very small between the PCR replicates for a same biological sample (Real-Time experimental replicate) compared to the variance between the different biological replicates. Calculations and statistical analyses were performed by ANOVA on the mean ΔC_T _(C_T _of each gene – C_T _of 18S RNA used as load control) of different biological replicates, as described in the Results section.

## Authors' contributions

MH designed the experiment, performed part of the experiments, analyzed the results and prepared the manuscript. AOD performed part of the experiments and their analysis, participated in preparing the figures and critically read the manuscript. All authors have read and approved the final manuscript.

## Supplementary Material

Additional file 1 **Microarray data of candidate stress-associated genes**. The differential expression between Al-treated and control (non-treated) samples was analyzed to identify genes that are significantly over-expressed two-fold or more. A first round of selection retained 70 genes for which there is at least two wheat lines with p values > 0.001 AND p values > 0.01 in the other two lines. A second round of selection for which there was at least three lines with p values > 0.001 retained an additional 13 genes (in yellow) for a total of 83 candidate genes. A = Atlas66; B = Bounty; C-T = Century-T; C-S = Century-S. Numbers associated with the line's abbreviation (0, 5 or 50) represents the Al concentration in μM while the following letter indicates the biological replicate sample number (S1 to S3). Click here for file

Additional file 2 **Microarray data of candidate tolerance-associated genes**. The differential expression between Al-treated tolerant and sensitive lines (A 50 – B 5 and C-T 50 – C-S 5) was analyzed to identify genes that are significantly over-expressed two-fold or more in tolerant compared to sensitive lines (ANOVA p values > 0.001 in one pair AND p values > 0.01 in the other pair of wheat lines). Genes on this list that were not differentially expressed between Al-treated and the controls (non-treated) are classified as constitutively expressed. A = Atlas66; B = Bounty; C-T = Century-T; C-S = Century-S. Numbers associated with the cultivar's abbreviation (0, 5 or 50) represents the Al concentration in μM while the following letter indicates the biological replicate sample number (S1 to S3). A: Constitutively expressed candidate genes associated with Al tolerance. B: Up-regulated candidate genes associated with Al tolerance. Click here for file

Additional file 3 **Primers used for amplification of different transcripts**. The transcripts representing the different probesetIDs boxed in Table 1 and Table 2 were used to design unique primers for qRT-PCR. Click here for file
