# Supplementary material for: Identification of genes and pathways associated with aluminum stress and tolerance using transcriptome profiling of wheat near-isogenic lines
Source: BMC Genomics. 2008 Aug 27;9:400. doi: 10.1186/1471-2164-9-400 (PMC2551624; doi:10.1186/1471-2164-9-400)
Supplement: Additional file 3 — Primers used for amplification of different transcripts. The transcripts representing the different probesetIDs boxed in Table 1 and Table 2 were used to design unique primers for qRT-PCR. [file 1471-2164-9-400-S3.doc]

Additional file 3- Primers used for amplification of different transcripts. The transcripts representing the different probesetIDs boxed in Tables 1 and 2 were used to design unique primers for qRT-PCR.

| ProbesetIDs^a^ | Annotation | Genbank | Forward primer (5’-3’) | Reverse primer (5’-3’) |
| --- | --- | --- | --- | --- |
| Ta.192.1.S1­_at | WCI-5 | U32431.1 | AGACCGGAGGGACTACGACT | CCTTCACCTCCTTCTTGACG |
| Ta.12921.1.S1._x_at | unknown | CA601406 | ACTGACCGATGATCCGAGAC | CCGCAGACGCAACACTAATA |
| Ta.5557.1.S1_x_at | Germin/ oxalate oxidase | CD869243 | TTCCCCCAGAACTGAGATTG | CAGCCAAAACCCTGGAGTTA |
| Ta.392.2.S1_at | unknown | CA659319 | AGGAAGGAACAGCGCAATAA | GCGAGATGGAAAACCCAATA |
| Ta.30659.1.S1_at | ALMT1 | AB081803.1 | ACACTTCTTGCGGACTTGGTTGATA | TTGTGTCCTCGGGGTTCTTA |
| TaAffx.16664.1.A1_at | F-box containing domain | CK205523 | ATACTCCCTGTTTTGCGGAATTGCC | TCGCAAAAGGTGTTGTCTCA |
| Ta.23271.1.S1_s_at | unknown | CA680274 | CTTACACCGCACCGCAAG | CATTTCCAGACACCCTTGACAAA |
| Ta.8545.1.S1_at | Glutathione S-transferase | BQ162041 | GAGCCGGTCATTGCGCTGCTGACTG | AGTGCAACTTCCCACTTTGG |
| Ta.21314.1.S1_x_at | disease resistance response protein | CA669694 | ACGACTGATGCATTGTCAGC | CCGAATAAATCATCGCCGTA |
| Ta.24632.1.S1_at | pathogen response | BE405372 | ATCGAGGAGGCCAAGAAGAT | AAAACGGTGGAGCATGAATC |

a: According to Affymetrix Gene Chip® wheat genome array description.
